# Large-scale computational identification of regulatory SNPs with rSNP-MAPPER

**DOI:** 10.1186/1471-2164-13-S4-S7

**Published:** 2012-06-18

**Authors:** Alberto Riva

**Affiliations:** 1 Department of Molecular Genetics and Microbiology, University of Florida, Gainesville, 32610, USA; 2 University of Florida Genetics Institute, University of Florida, Gainesville, 32610, USA

## Abstract

**Background:**

The computational analysis of regulatory SNPs (rSNPs) is an essential step in the elucidation of the structure and function of regulatory networks at the cellular level. In this work we focus in particular on SNPs that potentially affect a Transcription Factor Binding Site (TFBS) to a significant extent, possibly resulting in changes to gene expression patterns or alternative splicing. The application described here is based on the MAPPER platform, a previously developed web-based system for the computational detection of TFBSs in DNA sequences.

**Methods:**

rSNP-MAPPER is a computational tool that analyzes SNPs lying within predicted TFBSs and determines whether the allele substitution results in a significant change in the TFBS predictive score. The application's simple and intuitive interface supports several usage modes. For example, the user may search for potential rSNPs in the promoters of one or more genes, specified as a list of identifiers or chosen among the members of a pathway. Alternatively, the user may specify a set of SNPs to be analyzed by uploading a list of SNP identifiers or providing the coordinates of a genomic region. Finally, the user can provide two alternative sequences (wildtype and mutant), and the system will determine the location of variants to be analyzed by comparing them.

**Results:**

In this paper we outline the architecture of rSNP-MAPPER, describing its intuitive and powerful user interface in detail. We then present several examples of the use of rSNP-MAPPER to reproduce and confirm experimental studies aimed at identifying regulatory SNPs in human genes, that show how rSNP-MAPPER is able to detect and characterize rSNPs with high accuracy. Results are richly annotated and can be displayed online or downloaded in a number of different formats.

**Conclusions:**

rSNP-MAPPER is optimized for large scale work, allowing for the efficient annotation of thousands of SNPs, and is designed to assist in the genome-wide investigation of transcriptional regulatory networks, prioritizing potential rSNPs for subsequent experimental validation. rSNP-MAPPER is freely available at http://genome.ufl.edu/mapper/.

## Introduction

Single nucleotide polymorphisms (SNPs) have become the genetic marker of choice for studies aimed at linking genotype and phenotype, thanks to the availability of a very large number of well-characterized SNPs in public databases and of high-throughput technologies able to reliably genotype a large number of SNPs in parallel at a low cost. The most recent release of the dbSNP database, the largest public SNP repository [1], includes over 50 million human SNPs, the HapMap project has produced over 15 million SNP genotypes in individuals from eleven different populations [2], and several other efforts are under way to paint a comprehensive picture of genetic variation in humans.

As the number of SNPs that can be sampled in Genome-Wide Association Studies (GWAS) increases, there is a growing opportunity for using SNPs not simply as genetic markers, but also as causal candidates for diseases. Although the basic idea of GWAS is to look for a significant statistical association between a phenotype and one or more genetic markers in an unbiased way, knowledge of the potential functional roles of SNPs can greatly help in the design and interpretation of these studies. A drawback of the ability to genotype a very large number of SNPs offered by current high-throughput technologies is the high rate of false positives in the results [3]. Determining the potential functional role of a SNP on the basis of its precise location on the genome and of its alleles is therefore critical to our ability to prioritize candidate SNPs, distinguishing those worthy of further investigation from false positives.

In this work we focus on the large-scale computational identification of putative regulatory SNPs (rSNPs). We define rSNPs as those SNPs that potentially affect a Transcription Factor Binding Site (TFBS). By modifying the site's DNA sequence, a regulatory SNP can change the its binding affinity towards the corresponding TF, which in turn can have an effect on the expression level of the downstream gene. While coding SNPs amount to about 1% of currently known SNPs, out of which only a fraction cause an amino acid change, regulatory SNPs are expected to be much more numerous and to cause a wide range of biological and pathological consequences, since they can potentially affect the regulation, expression level and structure or function of the gene product [4]. If a method to reliably detect them is available, rSNPs could be linked with changes in gene expression levels or alternative splicing patterns, providing highly useful information for the elucidation of the corresponding regulatory networks.

Several online tools for the computational identification of putative regulatory SNPs exist. For example, the RAVEN application [5] determines the change in score produced by the two alleles of a DNA sequence containing a SNP when evaluated by a Probability Weight Matrix (PWM) representation of the binding site, while is-rSNP [6] uses convolution methods to determine the distribution of PWM scores corresponding to all possible nucleotide changes in a TFBS sequence, which helps in assessing the significance of an observed score change. Unfortunately neither of these systems is suitable for high-throughput work, and they both provide limited flexibility in the inputs they accept. RAVEN can only be used to search for regulatory SNPs in a single gene at a time, while is-rSNP provides the ability to analyze SNPs specified by the user or belonging to a specified chromosome region, but does not allow the user to search for SNPs on the basis of genes or other genomic elements.

The tool presented here is a component of MAPPER, a modular online application for the computational identification of TFBSs in DNA sequences [7,8]. MAPPER is based on an extensive library of probabilistic models representing several hundred TFBSs (generated by training a Hidden Markov Model on the multiple-sequence alignment of all known binding sites for each factor), and includes a *Database* of pre-computed putative binding sites in the promoter regions of three genomes, a *Search Engine* able to analyze a novel DNA sequence in real time, and *rSNP-MAPPER*, the tool described in this paper, designed to identify putative regulatory SNPs with high sensitivity in a high-throughput setting.

The MAPPER model library is divided into three groups of TFBS models. The first one (TRANSFAC models) was built using the same optimal alignments used to build the PWMs in the TRANSFAC database [9]. The second one (MAPPER models) is specific to MAPPER, and was built using TFBS sequences from TRANSFAC that were not used in the TRANSFAC models. Finally, the third library was generated using TFBS data from the JASPAR database [10]. Table 1 lists the number of models in each library and the number of TFs they represent. It should be noted that there may be multiple models for the same TF, due to the presence of different sets of binding sites for it in the source databases.

**Table 1:** Number of TFBS models and factors represented in the three default MAPPER libraries.

Library	Models	Factors
TRANSFAC	399	326
MAPPER	529	434
JASPAR	89	89
Total	1017	678

## Methods

### rSNP detection

MAPPER modules use Profile Hidden Markov Models trained on alignments of known sites to detect putative binding sites in DNA sequences. The *hmmpfam* program, from the HMMER package [11], is used to scan a DNA sequence with a library of models, and every putative binding site detected by it receives a numeric score that measures how well the site matches the TFBS model. RSNP-MAPPER identifies putative rSNPs by matching predicted TFBSs with the positions of known or user-specified SNPs, and evaluating the change in score caused by the allele substitution. The score change measures the potential impact of the SNP on the binding affinity of the site, and can therefore be used to prioritize candidate regulatory SNPs.

For example, the sequence “TAAAGTTTAGAT” is classified by MAPPER as a binding site for the HFH-1 transcription factor, with a score of 4.6. If a SNP was present in position 7 of that sequence changing the T to a C, the resulting sequence (“TAAAGT**C**TAGAT”) would receive a score of -0.2. Since a negative score indicates that the sequence is not considered a real HFH-1 binding site, rSNP-MAPPER would in this case predict that the T to C substitution effectively eliminates the HFH-1 binding site.

Given a set of SNPs, either specified by the user or generated using one of the methods described below, MAPPER automatically produces two alternative sequences, scans both of them with the desired models, and compares the resulting TFBSs. TFBSs that occur in both sets are ignored, since they are not affected by the SNPs. TFBSs that appear in both sets but with different scores (called *hit-pairs*), are instead collected and reported to the user. Hit-pairs are characterized by a *score change*, the absolute difference of the scores of the two matching TFBSs. Hit pairs are also generated for cases in which a TFBS occurs in only one set an not the other: in this case the score change is equal to the score of the known TFBS, since the score of the other one is unknown and is assumed to be 0. The score change therefore represents a low-bound estimate of the predicted impact of a SNP on a binding site

### User interface

**Figure 1:**
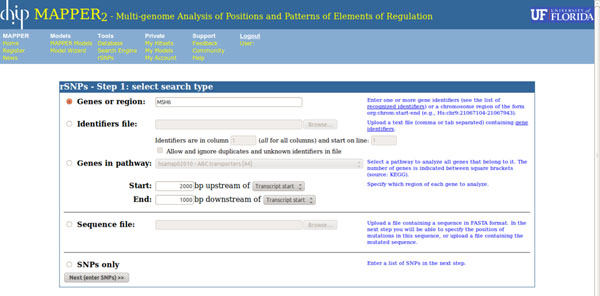
The first page of the rSNP-MAPPER interface, allowing the user to select the desired type of session. The form is divided into three parts: the top one is used to create sessions that analyze SNPs in one or more genes, the second one allows the user to upload a file containing a DNA sequence to be analyzed, and the third part indicates that the user is going to enter a list of SNP identifiers manually.

The rSNPs-MAPPER user interface was designed to provide the user with high flexibility and multiple modes of operation while remaining simple, intuitive, and consistent with the other MAPPER modules. The interface walks the user through the process of creating and executing an analysis session, which consists of three successive steps. The initial page, shown in Figure 1, allows the user to choose among six different ways of specifying the list of SNPs to be analyzed. They are:

1. Entering a chromosome region, in the form chrN:xxx-yyy, where N is the chromosome number, and xxx and yyy are the start and end coordinates of the region according to the most recent build of the human genome (currently, GRCh37/hg19).

2. Entering one or more gene or transcript identifiers, separated by commas, in a text field.

3. Uploading a file containing the gene or transcript identifiers.

4. Choosing a KEGG pathway from a menu, to select all genes in that pathway.

5. Uploading a file containing a DNA sequence (SNP locations are specified in the second step).

6. Entering a list of SNP identifiers.

In the case of options 2-4, the user can indicate the exact region of each gene to be analyzed by specifying its boundaries. The start of the region is specified as a number of nucleotides upstream of the transcript start, the start codon (ATG), the stop codon, or the transcript end, while the end of the region is specified as a number of nucleotides downstream of one of the same four reference points. For example, to analyze all SNPs inside a gene or within 2,000bp of it, the user would select 2,000bp upstream of the transcript start as the start position, and 2,000bp downstream of the transcript end as the end position. To analyze SNPs in the gene's promoter, the user could instead use 5,000bp upstream of the transcript start and 200bp downstream of the transcript start as the region boundaries.

After clicking the “Next” button the user is taken to a second page, whose exact contents depend on the method selected in the first step. If the genomic coordinates of the regions to be analyzed are known, as is true in cases 1-4, the interface provides the option to extract all known SNPs in those regions from the dbSNP database. The user can also indicate which allele should be used as the “wild type” allele; possible choices include: the reference allele (the one that appears in the reference genome sequence at that position), the ancestral allele (when known), or the major allele in one of the HapMap populations. Choosing the correct wild type allele improves the quality of the results, since it establishes the “direction” of a score change. The user should therefore select the HapMap population that most closely matches the one under study, if possible, or resort to the ancestral allele otherwise. The user may also enter mutations manually using either dbSNP “rs” identifiers or one of the notations “*pos*A>B” or “A*pos*B”, where A and B are the two alleles and *pos* is the SNP position, specified as an absolute genomic coordinate or as a distance from the start of the transcript or from the ATG. If the user submitted multiple genes/transcript for analysis, the text input field will be divided into multiple sections, one for each transcript.

In the case of option 5, SNPs can be specified either by entering them manually as described above (except that coordinates are always relative to the start of the uploaded sequence, and “rs” identifiers cannot be used), or by uploading a second DNA sequence of the same length. In the second case the two sequences will be compared base by base, and a SNP will be generated for each position where the two sequences do not match. In the case of option 6, the user should simply enter a list of “rs” identifiers in the text area.

Taken together, these options allow for a wide range of different analysis modes. Users may analyze genomic regions corresponding to known genes, arbitrary genomic regions, or DNA sequences from any source. Mutations can be specified by combining known SNPs from dbSNP with those specified by the user, either by entering their positions and alleles manually, or by providing wild type and mutant DNA sequences. The analysis session may consist of a single *run* (e.g. when a single genomic region is analyzed), or of multiple runs (e.g., when specifying a set of genes or sets of SNPs from different genomic regions). Finally it should be noted that, while all other options are currently restricted to human, option 5 can be used on DNA sequences from any organism.

The third and final page provides a summary of the session options specified so far, including the full, downloadable list of SNPs being analyzed, and allows the user to specify which TFBS models should be used in the analysis, and to provide an email address to be notified when the analysis is complete. The user can now start the session, if all parameters are correct, or return to the previous steps to modify them if necessary. The system will then display a page showing the progress of the session execution. Since runs are executed as background processes, the user is free to move to other pages and even create new sessions.

### Output

The output of rSNPs-MAPPER consists of a list of TFBSs whose score changes as a consequence of the presence of a SNP. Results are fully annotated and can be browsed, sorted, exported in various formats, displayed graphically, or uploaded to the UCSC Genome Browser as a “custom track” [12]. The page displaying the results is divided into two sections: a panel at the top providing information or commands, and a table listing all hit pairs. The contents of the panel are divided into five different groups of controls, that can be selected using the tabs at the top: *Summary*, *Inputs*, *Filters*, *Display*, and *Export*.

The *Summary* panel contains general information about the session being displayed, starting with its identifier, a brief description, and the total run time. The next line contains a menu allowing the user to select the run to be displayed, in case the session contains multiple runs (e.g., when analyzing all genes in a pathway), while the “Displayed sites” field shows the number of hit pairs displayed in the table, out of the total number of hit pairs produced. The *Inputs* panel provides information about the parameters of the run being displayed, including details about the gene or transcript provided as input, if any (symbol, name, mRNA accession, organism), the coordinates of the genomic region that was scanned, the full list of SNPs, and the model libraries used in the analysis.

The *Filters* panel provides commands to change the set of displayed hit pairs. Hit pairs can be selected based on the score of the hits they contain, either by entering a threshold value or by selecting a percentile level from a menu (since different models have different score distributions, this option allows the user to select high-quality hits independently of the actual numerical values of their scores). The score threshold applies to either one of the two hits in a hit pair: a hit pair is displayed if the score of either one of its two hits is above the threshold. The “Score change” field is instead used to filter hit pairs by putting a threshold on their score change. The combination of this threshold and the previous one allows the user to select high-quality TFBSs that are significantly affected by the presence of a SNP. Other options include selecting hit pairs on the basis of the name of the factor they apply to, and highlighting hits in evolutionarily conserved regions. The *Display* panel contains options to control how hit pairs are displayed in the main table: the user can specify the way hit pairs should be sorted (by position, score, score change, factor name, or factor accession), how hit coordinates are displayed (absolute on chromosome, relative to transcript start, relative to ATG), and whether only the best hit-pair for each SNP (i.e., the one with the highest score change) should be displayed instead of all of them. This option is provided to reduce the number of hit-pairs displayed giving priority to the TFBSs with the highest potential impact.

The *Export* panel provides controls to export the hit pairs in a variety of different ways. To start, the user may choose one of the available export formats: *text* (a delimited file with one row for each hit pair; *alignments* (similar to text, but including the hit alignments), *BED* (suitable for upload to the UCSC Genome Browser as a custom track), *GFF* (the General Feature Format defined by the Wellcome Trust Sanger Institute), and *image* (a graphical representation of the analyzed region showing the position and factor name for all hit pairs). The user can then specify the delimiter to use when generating files in *text* or *alignments* format (tab or comma character), the name of the file, whether the generated file should be compressed for faster downloads, and whether the system should produce a file containing hits for all runs in the current session, instead of those of the currently displayed run only. Finally, the user can choose whether to receive the exported hits as a downloadable file or by E-mail. This page also provides a link to automatically upload the results to the UCSC genome browser and to display them as a custom track.

The results table displays all hit pairs that are visible according to the settings in the *Display* panel. When the table is visible it displays the following fields: the SNP, using its “rs” identifier and/or the “A*pos*B” notation; the TFBSs scores corresponding to the two alleles, and the score difference; the model that produced the predicted TFBS and the corresponding transcription factor name; strand, chromosome, start and end position of the TFBS. In order to facilitate visual inspection of the results, score changes greater than 2 in either direction are highlighted, as they indicate that the alternative site is 100 times more likely (or less likely) to be real than the original one. While this should not be considered a true significance threshold, it is useful to quickly identify putative rSNPs with a potentially large effect.

**Figure 2:**
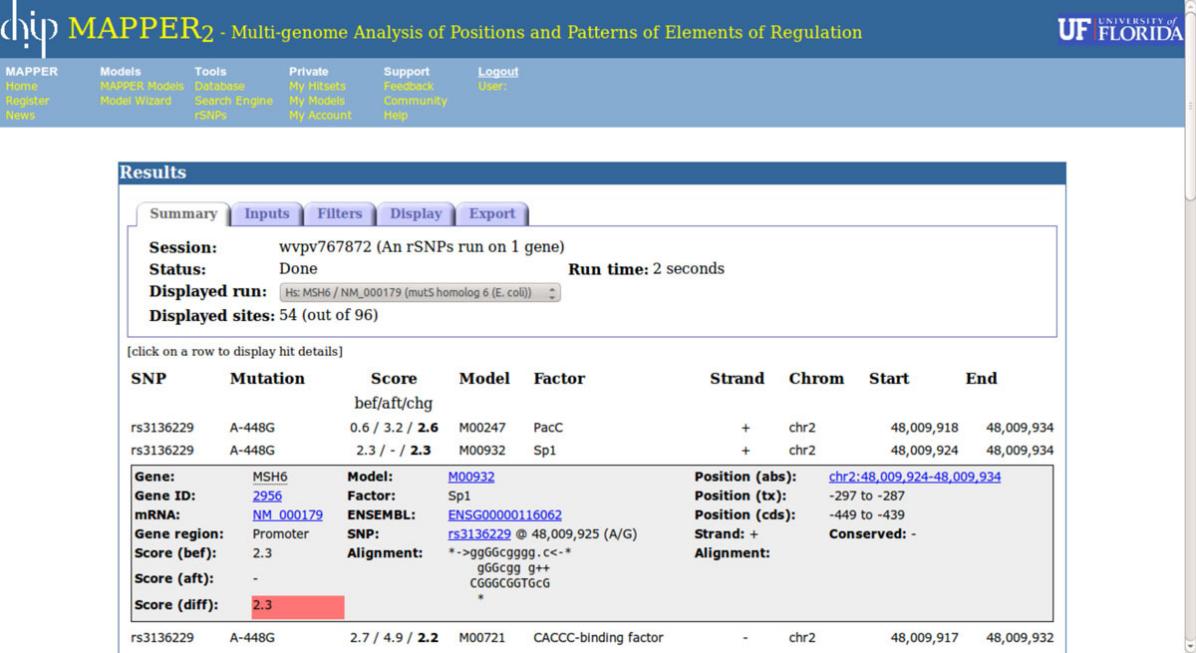
The page displaying the results of an rSNP-MAPPER run. The top part of the window displays run parameters and allows the user to control how results are displayed and exported. The bottom part contains the hit pairs produced by the program, one per line. Information shown for each hit pair includes the SNP identifier and position, the scores related to the two SNP alleles and their difference, the model that produced the predicted TFBS and the corresponding factor name, and the genomic coordinates of the TFBS.

Clicking the mouse button over a row opens a box containing more exhaustive information about the hit pair in that row. Additional fields displayed in this case include information about the gene, if any (gene symbol, NCBI gene id, ENSEMBL gene id, mRNA accession), the alignments corresponding to the two SNP alleles, the TFBS position according to all three reference systems (absolute on chromosome, relative to transcript start, relative to ATG), and a flag indicating whether the hit pair lies in an evolutionarily conserved region. Moreover, several fields in this box are hyperlinks to pages with further information. For example, the Gene ID is linked to the NCBI Gene page for that gene; the model accession number is a link to the MAPPER page describing that model, and the absolute hit position is a link to the UCSC Genome Browser. Figure 2 shows a typical result page for a single-gene rSNP-MAPPER run.

## Results

A direct comparison of rSNP-MAPPER against other tools for rSNP detection is difficult for at least two reasons: the lack of an up-to-date, publicly available, sufficiently large set of known rSNPs to be used as a test dataset, and the fact that, to the best of our knowledge, no other tool offers the kind of support for high-throughput rSNP analysis that is one of rSNP-MAPPER's strengths. To cite just one example, RAVEN only allows analyzing SNPs on a single gene at a time, its library of models is extremely limited, and the SNP database it relies on has not been updated in several years.

In this section we instead present several examples of rSNP-MAPPER runs, aimed at demonstrating the program's capabilities and usage. The first three examples are based on lists of known regulatory SNPs provided by two recent studies ([5] and [6]), while the next three deal with other sets of SNPs that were experimentally shown to affect gene transcription.

### Example 1: rSNPs from Andersen *et al*

We tested MAPPER on the 77 rSNPs from Dataset S1 in Andersen *et al*. [5] for which dbSNP 'rs' identifiers were available. 13 SNPs had to be excluded from the analysis because their identifiers were not found in MAPPER's database (for example, because they were withdrawn after publication of the original study). Of the remaining 64 SNPs, 51 (80%) produced score changes over the suggested threshold of 2 for at least one TFBS. Using a more relaxed threshold of 1.5, the number increases to 56 out of 64 (88%). The complete results of this test are available in additional file 1. The first column contains the SNP identifier, and each row represents a putative TFBS affected by a SNP according to rSNP-MAPPER predictions. The last column displays the score change caused by the SNP in the corresponding TFBS; score changes greater than or equal to 2 are in bold face. In general, multiple rows are present for each SNP because of overlapping TFBS predictions, and hits for the same SNP are reported in order of decreasing score change.

### Example 2: Prior Knowledge rSNPs from Andersen *et al*

We performed the same test on the set of 20 rSNPs for which the affected TF is known provided by Andersen *et al*. as Dataset S2 [5]. rSNP-MAPPER predicts score changes above the suggested threshold of 2 for 18 of them (90%), while if the threshold is lowered to 1.5 the number increases to 19 out of 20 (95%). In approximately half of the cases rSNP-MAPPER predictions include a binding site for the experimentally determined TF or a closely related one, although the exact number is difficult to determine due to differences in TF nomenclature. The complete results of this test are available in additional file 2 (the format of this file is the same as additional file 1).

### Example 3: Known disease-associated rSNPS from Macintyre *et al*

We tested rSNP-MAPPER on a list of 11 known disease-associated regulatory SNPs provided by Macintyre *et al*. as part of the description of their is-rSNP system (see Table 2 in [6]). rSNP-MAPPER predicts score changes above the suggested threshold of 2 for 10 out of 11 rSNPS (90%). In six of these cases, rSNP-MAPPER predictions show a significant effect on a binding site for the experimentally determined TF (with the same caveats regarding nomenclature mentioned in the previous example). The complete results of this test are available in additional file 3 (the format of this file is the same as additional file 1).

### Example 4: Gene MSH6

Seven functional Sp1/Sp3 binding sites were detected computationally and validated experimentally within the first 500 bps upstream of the ATG of the *MSH6* gene, and were shown to be required for its transcription. This region contains three known SNPs, two of which are contained in Sp1/Sp3 sites and may therefore affect them by abolishing or decreasing Sp-1 binding. The effect of these two polymorphisms on Sp-1 binding was experimentally demonstrated by gel-shift assays and luciferase expression following co-transfection of the transcription factor gene with reporter constructs containing the substitutions in CHO and human BJ skin fibroblasts [13].

rSNPs-MAPPER analysis indicates that SNP rs3136229 (A-448G) causes the deletion of a predicted Sp-1 site at -449 to -439 (G allele score: 2.3; A allele score: <0). The same allele change also appears to highly increase the strength of a putative PacC site at the same position, with a positive score change of 2.6. The second SNP, rs41540312 (C-159T), causes the deletion of a putative Sp-1 site at -168 to -158. This is confirmed by two different models for Sp-1, with score changes of 2.3 and 2.2 respectively. The same SNP causes a putative NRF-1 binding site to appear at the same position, again confirmed by two different models (score changes of 3.1 and 2.9 respectively).

This example demonstrates the program's ability to correctly detect experimentally validated rSNPs, and to complement them with additional predictions about the consequences of the allele substitutions they produce. While the effects on the the Sp-1 sites had been observed experimentally, rSNP-MAPPER was able to predict the binding of other transcription factors to the altered sites, potentially leading to further disruptions in the regulation of the *MSH6* gene.

### Example 5: Gene F9

The coagulation factor IX gene (*F9*) expresses a liver-produced protein that is an essential component of the coagulation cascade. Its promoter contains experimentally characterized binding sites for three TFs: CCAAT / enhancer binding protein (C/EBP), hepatocyte nuclear factor 4 (HNF-4) and androgen receptor (AR). Point mutations in these TFBSs are known to lead to various forms of hemophilia B: mutations in the C/EBP and HNF-4 sites lead to hemophilia B Leyden; mutations in the AR site lead to hemophilia B Brandenburg, characterized by a life-long deficiency in factor IX [14].

Since the mutations analyzed in this study do not appear in dbSNP they were entered manually in the rSNP-MAPPER interface, by specifying the allele change and the position relative to the start of the gene (e.g., A-26G). As shown in Table 2, rSNPs-MAPPER correctly identifies all the AR and C/EBP sites, and provides a measure of the effect of mutations on them. This example demonstrates rSNP-MAPPER's ability to analyze mutations that do not correspond to known SNPs, thus providing a tool for performing site-specific mutagenesis *in silico*.

**Table 2:** Analysis of 14 mutations linked to various forms of hemophilia in the Factor IX gene.

SNP	**Score** (bef / aft / change)	Model	Factor
A-26G	- / 4.9 / **4.9**	T00040	AR
C-26G	- / 4.9 / **4.9**	T00040	AR
C-23T	- / 4.9 / **4.9**	T00040	AR
G-21T	- / 4.9 / **4.9**	T00040	AR
C-20T	- / 2.4 / **2.4**	MA0044	HMG-1
A-20T	- / 2.4 / **2.4**	MA0044	HMG-1
G-19C	- / 1.7 / 1.7	MA0007	AR
T6A	- / 4.7 / **4.7**	MA0102	cEBP
T7C	- / 4.7 / **4.7**	MA0102	cEBP
T8C	- / 4.7 / **4.7**	MA0102	cEBP
C9G	- / 4.7 / **4.7**	MA0102	cEBP
A12C	- / 4.7 / **4.7**	MA0102	cEBP
A12G	- / 4.7 / **4.7**	MA0102	cEBP
A14G	- / 4.7 / **4.7**	MA0102	CEBP

### Example 6: Gene COL18A1

Type XVIII collagen (*COL18A1*) is a homotrimeric basement membrane molecule of unknown function, whose COOH-terminal NC1 domain contains endostatin (ES), a potent antiangiogenic agent [15]. Different levels of *COL18A1* expression have been associated with several pathological processes such as cancer, liver fibrosis, diabetic retinopathy and Alzheimer’s disease. Studies focused on the characterization of cis-regulatory elements interacting with human *COL18A1* promoter 2 and the identification of SNPs in this region in different ethnic groups have reported that SNP rs755548 (T-700G) is associated with an increase in transcription and binding for an unknown transcription factor for the -700G allele in hepatocytes [16].

rSNP-MAPPER analysis predicts that SNP rs755548 affects a putative site for either the POU2F2 or the ETS transcription factors. Moreover, it shows that SNP rs3810589, occurring in close proximity to the former, may also have a significant effect on the binding of ZID, BSAP, ER-alpha TFs. This example shows how rSNP-MAPPER can be used to generate hypotheses about the effect of a known regulatory SNP on TF binding, even when the TF is not known in advance.

## Conclusions

rSNP-MAPPER is a powerful tool for the large-scale analysis of regulatory SNPs. Its main purpose is to evaluate the potential effect of a SNP on predicted transcription factor binding sites, by measuring the change in the predictive score of the TFBS as a consequence of the allele change. Large score changes indicate that the SNP has the potential to significantly affect the site's affinity for its binding factor, leading to possible disruptions of the regulatory network. rSNP-MAPPER can therefore be used to generate hypotheses on potential regulatory SNPs, by screening all SNPs in a genomic region of interest, or to validate experimental results, by analyzing a specific set of SNPs.

rSNP-MAPPER is a web-based application freely available at http://genome.ufl.edu/mapper/. The system can be used in *guest* mode, but creating an account is recommended. Accounts are free, and provide users with the ability to store the results of their analyses in a private area of the MAPPER server.

rSNP-MAPPER is designed to efficiently perform high-throughput analysis, giving the user the ability to analyze hundreds of genes and thousands of SNPs at once (there is no set limit to the size of a run, except for the constraints imposed by the hardware the system is hosted on). Its simple and intuitive interface supports multiple modes of operation: it can be used to analyze SNPs located within genes or close to them, SNPs in a specified genomic region, or a user-specified set of SNPs. The output of the program is fully annotated and can be exported or displayed in several different formats, in order to facilitate subsequent analysis and validation of the results.

rSNP-MAPPER implements a highly accurate rSNP analysis method, thanks to the use of a powerful TFBS-detection method based on Hidden Markov Models rather than Probability Weight Matrices. In addition to the the examples presented in the Results section, we have tested it on the experimentally-validated regulatory SNPs available in the HGMD database with excellent results. Further strengths of rSNP-MAPPER include its very extensive library of binding site models, and a powerful and flexible user interface that cover most analysis needs, ranging from single-gene searches to high-throughput analysis of thousands of SNPs.

## List of abbreviations used

GWAS: Genome-Wide Association Study; PWM: Probability Weight Matrix; SNP: Single-Nucleotide Polymorphism; rSNP: Regulatory SNP; TFBS: Transcription Factor Binding Site.

## Competing interests

The author declares no competing interests.

## Supplementary Material

Additional file 1 **rSNP-MAPPER results on the data provided by Andersen *et al*.** Excel spreadsheet showing the results of rSNP-MAPPER analysis on 64 putative regulatory SNPs from Andersen *et al *[5]. Click here for file

Additional file 2 **rSNP-MAPPER results on the data provided by Andersen *et al*.** Excel spreadsheet showing the results of rSNP-MAPPER analysis on 20 known regulatory SNPs from Andersen *et al *[5]. Click here for file

Additional file 3 **rSNP-MAPPER results on the data provided by Macintyre *et al*.** Excel spreadsheet showing the results of rSNP-MAPPER analysis on 11 disease-associated regulatory SNPs from Macintyre *et al *[6]. Click here for file
